# Reference intervals established using indirect method for serum ferritin assayed on Abbott Architect *i*2000_SR_ analyzer in Chinese adults

**DOI:** 10.1002/jcla.23083

**Published:** 2019-11-01

**Authors:** Qing‐ping Wang, Lin‐ying Guo, Zhi‐yong Lu, Jian‐wen Gu

**Affiliations:** ^1^ Department of Clinical Laboratory The Shaoxing Hospital of China Medical University Shaoxing China; ^2^ Department of Health Examination Center The Shaoxing Hospital of China Medical University Shaoxing China; ^3^ Department of Clinical Laboratory The Third Affiliated Hospital of Suzhou University Changzhou China

**Keywords:** adults, Chinese, indirect method, reference intervals, serum ferritin

## Abstract

**Background:**

Serum ferritin (SF) test has been widely used in clinical practice. However, its reference intervals (RIs) vary depending on the analytical method and ethnic origin. This study was to establish the RIs using indirect method for SF in Chinese adults.

**Methods:**

SF was assayed on Abbott *i*2000_SR_ analyzer. The SF test results of all health examinees (8913 males aged 18‐93 years and 5397 females aged 18‐90 years) between December 2010 and April 2019 were obtained from our laboratory information system. After *Box‐Cox* transformation of raw data and exclusion of outliers, parametric and non‐parametric approaches were used to calculate 95% RIs. The correlation between SF levels and ages, and the differences in SF levels between subgroups were also analyzed.

**Results:**

SF levels in females were significantly different from those in males (*Z* = 88.96, Z* = 23.17; *Z* > Z*) and showed a weak positive correlation with age (*r* = .466, *P* < .0001). The RIs based on parametric approach in males were 66.12‐561.58 µg/L, whereas in all females were 3.59‐269.59 µg/L, females aged <50 years 3.26‐148.02 µg/L and those aged ≥50 years 17.28‐303.27 µg/L. The RIs based on non‐parametric approach in males were 65.00‐571.37 µg/L whereas in all females were 4.00‐254.00 µg/L, females aged <50 years 4.00‐152.00 µg/L and those aged ≥50 years 16.00‐304.05 µg/L.

**Conclusions:**

Our indirect RIs for SF were markedly different from the manufacturer's recommended RIs and might be more suitable for Chinese adults, which would be helpful in interpreting laboratory data and clinical decision‐making.

## 1 INTRODUCTION

Ferritin, a soluble 450 kDa protein, is a major iron storage protein presenting in the intracellular compartments.^1^, ^2^, ^3^ As the most abundant transition metal element in the body, iron is widely involved in various physiological processes such as oxygen transport, electron transport, cell cycle regulation, energy metabolism, and DNA synthesis.^4^, ^5^ Ferritin provides intracellular storage of bio‐available iron in a safe and readily accessible form and protects cells from iron‐mediated free radical formation and toxicity^1^, ^3^. Normally, a small amount of ferritin exists in the peripheral circulating blood and is called serum ferritin (SF). Under normal circumstances, the amount of ferritin synthesized and secreted into the serum is proportional to the amount of cellular ferritin produced in the internal iron storage pathway, so that SF concentration is usually related to the quantity of body iron stores. Clinically, SF has been reported to link with iron‐deficiency anemia or a risk of iron overload.^6^, ^7^ For these reasons, SF is widely used as a surrogate marker of iron stores. In addition to this, SF has been found to be elevated in patient with inflammation, liver disease, and malignancy.^8^, ^9^, ^10^, ^11^ Moreover, previous studies have suggested that high SF is associated with poor prognosis in pancreatic cancer, colorectal cancer, lung cancer, peripheral T‐cell lymphoma, and hepatocellular carcinoma.^12^, ^13^, ^14^, ^15^, ^16^ Therefore, ferritin may not only function as a marker of iron stores, but may also aid in clinical decision‐making, including diagnosis, prognosis, treatment, and/or patient management. As a result, the number of tests for ferritin increase rapidly year by year.^17^

As the foundation for the interpretation of medical laboratory data, reference intervals (RIs) for analytes are one of the most widely used clinical decision‐making tool.^18^ Nevertheless, although measurement of SF has largely replaced laboratory assays of serum iron and transferrin or total iron‐binding capacity in clinical practice, there is no gold‐standard method for measuring SF. At present, SF can be measured using immunoassays, for example, enzyme‐linked immunosorbent assay (ELISA), electrochemiluminescent immunoassay (ECLIA), chemiluminescent immunoassay (CLIA), or immunoturbidometric assay.^3^, ^19^ Because of diversity and heterogeneity of ferritin antigens, and issues related to the reagent design in different assay methods, the RIs or normal ranges for SF in individuals vary depending on the assay method used.^19^, ^20^, ^21^ This makes it difficult for physicians to interpret the measurement results. In addition, studies have addressed there are significant ethnic‐specific differences in SF levels.^3^, ^22^ Hence, it is necessary to establish their own RIs for use in clinical laboratories with their population and analytical methods.^18^

According to international recommendations by the International Federation of Clinical Chemistry (IFCC) and the Clinical and Laboratory Standards Institute (CLSI),^23^ the so‐called “direct method,” in which healthy individuals representing the reference population are selected and sampled and the specimens from reference population are analyzed, is preferred for establishing RIs. In the “direct” method, a major problem that the laboratory faces is to obtain a sufficient number of specimens from healthy individuals representative of the reference population that the laboratory serves.^24^ Thus, as an alternative method, the “indirect” method via large collections of laboratory data stored in laboratory information systems (LIS) and statistical program is widely used to establish the RIs in recent years.^24^, ^25^, ^26^, ^27^, ^28^, ^29^, ^30^ Relative to the direct method, the “indirect” method is much simpler, faster, and cheaper^25^ and therefore is a convenient way for the clinical laboratory to set up their own RIs. In previous studies, however, most of which focused on the data obtained from hospital patient records and a group of “healthy” subjects was selected as the reference population from these hospital patients based on some statistical criteria,^24^, ^27^, ^28^, ^29^, ^30^ very few of which determined the RIs for analytes using the data from hospital database of health examination records. Although these data may not represent the general population, subjects were those who visited the health examination center for regular check‐ups, but not for treatment of a confirmed disease, which may lower the selection bias compared with previous hospital‐based studies. Therefore, the objective of the present study was to establish the RIs for SF, which is assayed by chemiluminescent microparticle immunoassay (CMIA) on the Abbott Architect *i*2000_SR_ analyzer, by an indirect method using the data from our LIS records of health examinees.

## 2 MATERIALS AND METHODS

### 2.1 Data source

All raw data were obtained from the database of the LIS of Shaoxing Hospital of China Medical University (Shaoxing, Zhejiang, China PR). Except for pregnant women, all subjects aged ≥18 years who had undergone SF testing during their visiting the health examination center for routine check‐up between December 2010 and April 2019 would be considered eligible for this study. Data were excluded from the study without any statistical analysis if the SF levels >2000 or <1 µg/L because these values exceeding the detection limits of the assay methods. Data with missing demographic information were also removed. In addition, undesirable duplicate test results were removed by data cleaning technology, only the first result for each subject (a unique code, eg, medical record number, is preferred to use recognizable identifiers) was retained for further analysis.

This study was approved by the institutional ethics review board of the Shaoxing Hospital of China Medical University (Shaoxing, Zhejiang, China PR, ethical approval number: 20190429) and was in accordance with the Helsinki Declaration of 1975 (revision 1983).

### 2.2 SF measurement and quality control

Venous blood samples were obtained from each study participant after a 12‐hours fasting period and collected into BD vacutainer tubes (Becton Dickinson). Serum was separated from the blood by centrifugation at 3000 rpm for 5 min at room temperature (18‐25°C). Specimens with hemolysis, icterus, and lipemia were excluded according to the rules of the laboratory. All specimens were quantitatively determined within 2 hours of collection by CMIA on Abbott Architect *i*2000_SR_ analyzer (Abbot Diagnostics) according to the manufacturer's specifications and following the laboratory standard operating procedures. The detection kits and calibrators were provided by Abbott Diagnostics. To ensure the accuracy and reliability of the analysis, assays were traceable to the 3rd international recombinant standard for ferritin (National Institute for Biological Standards and Control Code 94/572) and subject to participate in internal quality control (IQC) at different levels every day and inter‐laboratory external proficiency testing (PT) schemes from local external quality assessment (EQA) provider (Zhejiang, China) every year. Throughout this study period, the total analytical coefficient of variation (CV) was <10% and the PT/EQA score was more than 80% for SF, which met the quality assurance requirements as described in the Clinical and Laboratory Standards Institute's (CLSI) EP15‐A3 guidance document.^31^

### 2.3 Establishment of RIs for SF

All data were analyzed using EXCEL and MedCalc Statistical Software version 15.2.2 (MedCalc Software; http://www.medcalc.org; 2015). The establishment of RIs for SF in the present study would involve outlier removal, either before or after transformation of data, followed by calculation of the mean and standard deviation (SD) or median and relevant percentiles. Briefly, the steps are as follows:

#### 2.3.1 Evaluation of test results’ distribution and transformation of data

Skewness‐Kurtosis test was used to evaluate whether the given test results are distributed symmetrically or normally or whether the distribution is asymmetrical or skewed.^32^ When the data were significantly skewed, *Box‐Cox* would be used to transform the data to approximately normal or symmetrical distribution. The *Box‐Cox* transformation is defined as:

$$x\left(\lambda \right)=\left(x^{\lambda }-1\right)/\lambda \;\text{when}\;\lambda \neq 0.$$

x(λ)=ln(x)whenλ=0.

where *λ* is an undetermined parameter, which is obtained by the maximum likelihood method.

#### 2.3.2 Deletion of outliers

In this study, *Tukey* methods were used to check for multiple outliers at either side. In this method, an outlier value is defined as a value that is smaller than the lower quartile minus 1.5 times the interquartile range (IQR, the range from the 25th to the 75th percentile), or larger than the upper quartile plus 1.5 times the IQR. The calculation formulas are below:

Lowerlimit=P25-1.5×IQR

Upperlimit=P75+1.5×IQR

With Tukey's criterion, an outlier will be eliminated and this process is repeated on the remaining data until all outliers are eliminated. When the data are significantly skewed, the outliers are removed after *Box‐Cox* transformation. Following the outlier removal, the remaining data are re‐transformed and the outliers are removed again (iteration) until no outlier after the final data transformation. Then, the data were back‐transformed to the original values for presentation.

#### 2.3.3 Determination of RIs

Parametric or non‐parametric percentile statistical approach as described in the CLSI guidelines EP‐A3 guidelines can be used to calculate RIs in indirect method.^21^, ^24^, ^25^, ^33^ For parametric approach, the 95% RIs (double‐sided) was defined mean ± 1.96 SD; for non‐parametric approach, the values of the 2.5th and 97.5th percentile were used as lower and upper limits of RIs following the guideline EP28‐A3,^23^ respectively. Meanwhile, the 90% confidence intervals (CI) for lower and upper limits were also calculated. The RIs were also established for the various age‐ and sex‐specific subgroups when there are significant differences between the subgroups. Rank correlation was used to analyze the correlation between the SF levels and age. The standard normal deviation test (*Z* test) was performed to reveal the significance of differences in SF levels between the subgroups,^23^, ^34^ and the calculated *Z* > critical *Z* (Z*) was considered to be different from each other between subgroups.

## 3 RESULTS

A total of 15 949 raw data for males and 8141 for females were obtained from our LIS database, respectively. Of which, 7036 data for males (44.12%) and 2744 for females (33.70%) were excluded either because of the SF levels exceeding the detection limit of the methodology or because of missing information or repeated results. Finally, 8913 males aged 18‐93 years and 5397 females aged 18‐90 years were included in this study for further analysis.

**Figure 1:**
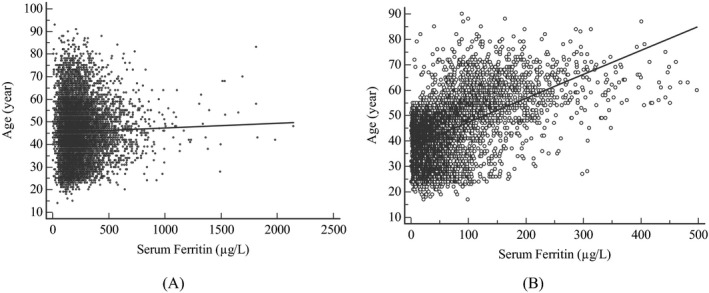
Correlation between age and serum ferritin levels. A, Males; B, Females

The statistical significance of the difference between SF in males and in females was tested by the *Z* test, and significant difference was found between males and females (*Z* = 88.96, Z* = 23.17; *Z* > Z*). Thus, 95% RIs and their 90% CIs were established separately for males and females. Rank correlation analysis showed SF levels had a weak positive correlation with age in females (Spearman's coefficient of rank correlation (*ρ*) = 0.466, *P* < .0001) but no significant correlation between SF and age was found in males (Spearman's coefficient of rank correlation (*ρ*) = 0.0193, *P* = .0682) as shown in Figure 1. *Z* test revealed there was a significant difference in SF levels between females aged <50 years and those aged ≥50 years (*Z* = 34.71, Z* = 14.22; *Z* > Z*). So, we established the age‐specific RIs and their CIs for females finally.

**Figure 2:**
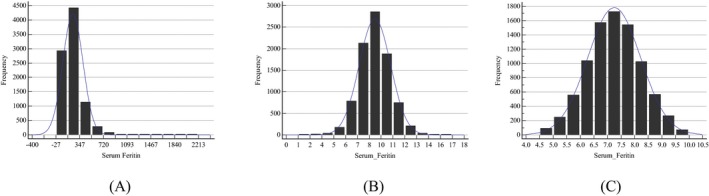
Frequency distribution graphs of serum ferritin (SF) for males. A, Data prior to transformation. B, Data after Box‐Cox transformation. C, Data excluding outliers after Box‐Cox transformation

**Figure 3:**
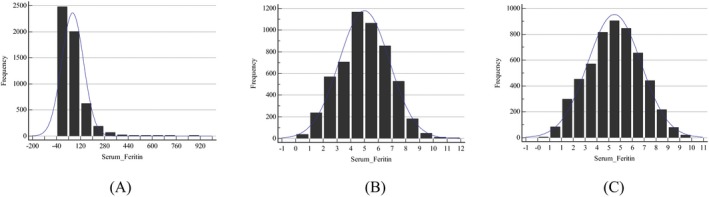
Frequency distribution graphs of serum ferritin (SF) for all females. A, Data prior to transformation. B, Data after Box‐Cox transformation. C, Data excluding outliers after Box‐Cox transformation

In this study, the SF in either males or females or their subgroups showed a significant skew distribution when Skewness‐Kurtosis test was used. After *Box‐Cox* was used to transform the data, all of them were approximately normal or symmetrical. The SF level distributions of sex‐ and age‐specific subgroups are detailed in Table 1, and the representative histograms are shown in Figures 2 and 3. After at least 2 iterations, the outliers in each group or subgroups were removed completely. The number of iterations and outlets in each group or subgroups is presented in Table 2. Then, we established 95% RIs for males and females and their age‐specific subgroups based on parametric and non‐parametric approach (Table 3). Compared with the manufacturer's recommended RIs, the RIs established for all females showed a slight difference; however, the RIs established for the other subgroups, whether based on parametric approach or based on non‐parametric approach, were markedly different (Table 3).

**Table 1:** Skewness‐Kurtosis test before or after Box‐Cox transformation in various subgroups

	n	Before transformation				After transformation			
		CS	*P* value	CK	*P* value	CS	*P* value	CK	*P* value
Outliers undeleted									
Males	8913	2.7256	<.0001	15.7791	<.0001	0.03184	.2195	1.1574	<.0001
All females	5397	2.6969	<.0001	12.4985	<.0001	0.009027	.7864	−0.389	<.0001
Females aged <50 y	3758	2.4131	<.0001	8.9486	<.0001	−0.01413	.7232	−0.2771	.0001
Females aged ≥50 y	1639	1.9839	<.0001	7.3882	<.0001	0.01375	.8196	0.5268	.0004
Outliers deleted									
Males	8704	1.2173	<.0001	1.6184	<.0001	0.000449	.9863	−0.2635	<.0001
All females	5385	2.0562	<.0001	5.527	<.0001	−0.02546	.4451	−0.4779	<.0001
Females aged <50 y	3746	1.9337	<.0001	4.9303	<.0001	−0.02589	.5171	−0.3855	<.0001
Females aged ≥50 y	1558	0.898	<.0001	0.629	.0001	−0.04441	.4728	−0.309	.0036

Abbreviations: CK, Coefficient of Kurtosis; CS, Coefficient of Skewness.

**Table 2:** Number of iterations and outliers in different subgroups

Subgroups	Sample size	Number of iterations	Number of outliers
Males	8913	3	209
All females	5397	2	12
Females aged <50 y	3758	2	12
Females aged ≥50 y	1639	2	81

**Table 3:** 95% Reference intervals of serum ferritin for males and females

	Established RIs based on parametric method			Established RIs based on non‐parametric method			Recommended Rls (µg/L)
	RIs (µg/L)	90% CI for LL	90% CI for UL	RIs (µg/L)	90% CI for LL	90% CI for UL	
Males	66.12‐561.58	64.87‐67.39	553.38‐569.89	65.00‐571.37	63.00‐67.00	561.00‐582.00	21.18‐274.66
All females	3.59‐269.59	3.38‐3.81	261.22‐278.19	4.00‐254.00	4.00‐4.00	244.13‐268.00	4.63‐204
Females aged <50 y	3.26‐148.02	3.05‐3.48	143.24‐152.94	4.00‐152.00	3.00‐4.00	143.00‐156.00	
Females aged ≥50 y	17.28‐303.27	15.60‐19.07	293.94‐312.77	16.00‐304.05	14.00‐17.00	291.00‐320.00	

Abbreviations: LL, lower limits; RIs, Reference intervals; UL, upper limits.

## 4 DISCUSSION

In this study, we established the RIs for SF in Chinese adults using the data obtained from our LIS and found that the RIs for SF in Chinese adults were markedly different from the manufacturer's recommended RIs.

Reliable and accurate RIs for laboratory analyses are an integral part of the process of correct interpretation of clinical laboratory test results. RIs given in laboratory reports have an important role in assisting the clinical decision‐making, including diagnosis, prognosis, treatment, and/or patient management.^18^ However, to produce high‐quality RIs according to the recommendations of IFCC for all relevant analytes is far beyond the capacity of a single laboratory. Therefore, many laboratories adopt RIs from other laboratories or use manufacturer's recommended RIs directly. Nevertheless, the RIs for most analytes are to some degree method‐ and instrument‐dependent.^19^, ^20^ In addition, differences between populations are also an issue and can reduce the validity of RIs.^3^, ^35^ For example, it was reported that one method can give 1.63 times higher values than the other method for SF^19^; mean SF values are found to be higher at all ages in adult black males than in adult white males.^35^ For these reasons, scientists have investigated the possibility via large collections of laboratory data for the goal of determining population RIs by an indirect method. So far, various indirect methods for establishing RIs have been developed.^24^, ^27^, ^28^, ^29^, ^30^ These methods may be used for the selection of a group of healthy subjects from a general hospital population, and RIs are calculated from hospital data using statistical approaches. In the present study, we attempted to establish the RIs for SF in Chinese adults using the data available from our LIS records. Unlike the data in most of the previous studies, the data in the present study were obtained from the subjects during their visit for the health examination center. The very low probability of disease makes these subjects obviously more desirable as the reference population than hospital patients since exclusion of extreme results, which are those most likely to be affected by disease, is a vital issue in establishing RIs.^24^, ^27^, ^28^, ^29^, ^30^

The present study demonstrated the SF levels had a significant difference between females and males, and had a positive correlation with age in females. Nevertheless, the manufacturer simply recommends 21.18‐274.66 µg/L as the RIs for adult males and 4.63‐204 µg/L for adult females but does not recommend the age‐specific RIs. To gain insight into the effect of age on SF levels, we further tested whether there was a significant difference between various age‐specific subgroups by *Z* test and found that the SF levels in females aged ≥50 years were significantly higher than those in those aged <50 years. These findings are in good agreement with those by Ogilvie et al^36^ and Adams et al.^37^ Indeed, SF values in adult females start to rise after 50 years of age as a result of iron loss from menstruation and pregnancies.^3^ In view of this, it is necessary to establish the sex‐ and age‐specific RIs for SF in our own laboratory.

In the indirect method, RIs can be calculated using various statistical approaches.^25^ Of these statistical approaches, the standard parametric or non‐parametric percentile approach is usually used in the direct method for RIs as described in the CLSI EP‐A3 guidelines.^23^ Jones et al^25^ suggested that the standard parametric or non‐parametric statistical approach can be used in the indirect method for RIs when the data were collected at a community screening project or similar. In fact, the parametric approach has been used in the analysis of data from NHANES,^21^ and the non‐parametric approach has been used in a Chinese and a Turkish study, respectively.^24^, ^33^ Of course, when the standard parametric approach is used, this will involve data transformation if the data are non‐normal or asymmetric distribution. In this study, our data showed a significant skew distribution but were symmetrical or approximately normal distribution after transformation. Therefore, we established the RIs for SF based on parametric and non‐parametric approach, respectively, and found that there was no significant difference between these two approaches for establishing RIs. Interestingly, our study demonstrated both the lower and the upper limits of RIs in Chinese adult males were markedly higher than those of manufacturer's recommended Rls. In Chinese females, those aged over 50 years also had elevated lower and upper limits of RIs for SF. Even in whole Chinese adult females, the upper limits of RIs for SF are also elevated. Similar findings were observed in the Canadian Health Measures Survey by Khosrow et al.^38^ As mentioned above, these findings might be related to considerable variation in SF levels in ethnic origin.^3^, ^22^, ^35^ In fact, in multi‐ethnic population studies in the USA, Adams et al^39^ had proved that elevated SF levels are more frequent in Afro‐Caribbean and Asian subjects than in whites or Hispanics. In a previous Chinese study on Cobas® 6000 system E601 (Roche) analyzer, Li et al^40^ also found that Chinese adult males had higher RIs for SF. However, compared with that study,^40^ our study had slightly but significantly lower RIs for SF in Chinese adult males. This may be related to the different assay methods used, as reported in previous studies.^19^, ^20^, ^21^ Taken together, we believe that the sex‐ and age‐specific RIs for SF established in this study are appropriate.

In conclusion, the present study has provided evidence that the RIs for SF in Chinese adults are markedly different from the manufacturer's recommended Rls. In Chinese adult females, the age‐specific RIs must be also taken into account. As a result, the establishment of the sex‐ and age‐specific RIs by indirect method using the data from health examination records for SF in our own laboratory would give a better chance to aid the physician in differentiating or considering treatment of iron‐deficiency anemia or a risk of iron overload than using manufacturer's recommended RIs or adopting RIs from other laboratories.
